# Hypoxia and Hypoxia-Inducible Factors in Lymphedema

**DOI:** 10.3389/fphar.2022.851057

**Published:** 2022-03-28

**Authors:** Xinguo Jiang, Wen Tian, Dongeon Kim, Alexander S. McQuiston, Ryan Vinh, Stanley G. Rockson, Gregg L. Semenza, Mark R. Nicolls

**Affiliations:** ^1^ VA Palo Alto Health Care System, Palo Alto, CA, United States; ^2^ Stanford University School of Medicine, Stanford, CA, United States; ^3^ Departments of Pediatrics, Medicine, Oncology, Radiation Oncology, and Biological Chemistry, and McKusick-Nathans Institute of Genetic Medicine, Vascular Program, Institute for Cell Engineering, Johns Hopkins University School of Medicine, Baltimore, MD, United States

**Keywords:** HIF, lymphedema, inflammation, lymphangiogensis, hypoxia

## Abstract

Lymphedema is a chronic inflammatory disorder characterized by edema, fat deposition, and fibrotic tissue remodeling. Despite significant advances in lymphatic biology research, our knowledge of lymphedema pathology is incomplete. Currently, there is no approved pharmacological therapy for this debilitating disease. Hypoxia is a recognized feature of inflammation, obesity, and fibrosis. Understanding hypoxia-regulated pathways in lymphedema may provide new insights into the pathobiology of this chronic disorder and help develop new medicinal treatments.

## Introduction

Lymphedema manifests as an initial accumulation of extracellular fluid and heightened inflammation with progressive chronic tissue remodeling characterized by fat deposition and fibrosis (Jiang et al., 2018; Rockson et al., 2019; Li et al., 2020). In the United States, lymphedema afflicts more than five million patients, most of them being cancer survivors who have undergone lymph node removal or radiotherapy (Rockson, 2018; Kataru et al., 2019). Current treatments for lymphedema are physiotherapy and compression garments, but these interventions only transiently reduce edema being ineffective in preventing or reversing disease progression (Rockson, 2008). There is an urgent need to improve the understanding of lymphedema pathogenesis and develop pharmacological therapies.

Prior research demonstrated that hypoxia regulates tumor lymphangiogenesis by promoting lymphatic endothelial expression of Prox-1 and pro-growth molecules such as VEGFC/VEGFR3 and CXCL12/CXCR4 (Irigoyen et al., 2007; Zhou et al., 2013; Ji, 2014; Morfoisse et al., 2015). Emerging precinlinal research indicates that lymphedema tissues are hypoxic with metabolic abnormalities (Poeze, 2006; Tabibiazar et al., 2006; Zampell et al., 2012a; Jiang et al., 2020). These studies suggest that hypoxia-regulated signaling may play a crucial role in lymphedema progression through modulating lymphangiogenesis.

Research over the last 15 years implicates inflammation as a crucial factor promoting lymphedema (Tabibiazar et al., 2006; Ly et al., 2017; Azhar et al., 2020). Clinical studies demonstrated lymphedema tissues are inflamed (Lin et al., 2012). SNP analysis revealed inflammatory gene variants that increase the risk of lymphedema in patients following breast cancer surgery (Leung et al., 2014). Immune cell profiling has revealed that lymphedematous tissues exhibit increased CD4^+^ T cells, including Th1, Th2, Th17, and regulatory T cells (Tregs) (Avraham et al., 2013; Gousopoulos et al., 2016; Ogata et al., 2016). Th2 cells aggravate lymphedema by augmenting tissue fibrosis (Avraham et al., 2013; Ly et al., 2019), whereas Th1 and Th17 cells promote non-productive lymphangiogenesis and exacerbate lymphedema by enhancing macrophage VEGFC production (Ogata et al., 2016). Tregs are beneficial but insufficient in suppressing effector cells in lymphedema (Gousopoulos et al., 2016). Innate immunity also contributes to lymphedema pathogenesis (Ly et al., 2017; Jiang et al., 2018; Azhar et al., 2020). For instance, dendritic cells (DCs) activate T cells and promote lymphedema (Li et al., 2020); macrophages exert context-dependent effects on lymphedema pathophysiology (Zampell et al., 2012b; Ghanta et al., 2015); and the myeloid cell-derived lipid mediator leukotriene B_4_ (LTB_4_) promotes lymphedema and blocking LTB_4_ production reverses the disease (Tian et al., 2017). Prolonged inflammation and edema ultimately induce chronic tissue pathology including excessive fat accumulation and fibrosis that are irresponsive to physiotherapy and compression garments (Jiang et al., 2018; Jiang et al., 2019a; Azhar et al., 2020; Li et al., 2020).

Anti-inflammatory therapies, including ketoprofen (NSAID) and Th2 cell inhibition, improve skin pathology but are ineffective in reducing limb volume and fat deposition (Rockson et al., 2018; Mehrara et al., 2021). Findings from these pilot clinical trials suggest that anti-inflammatory therapy alone may not be sufficient to reverse lymphedema. A better understanding of the mechanisms involved in inflammation, tissue fibrosis, and adipose expansion will provide a fuller view of lymphedema pathogenesis. As tissue hypoxia and cellular responses to hypoxic stress regulate immune function, adipose pathology, and fibrosis in various chronic diseases (Semenza, 2003; Trayhurn et al., 2008; Eltzschig and Carmeliet, 2011; Trayhurn, 2013; Manresa et al., 2014; Taylor and Colgan, 2017; Lee et al., 2019; Semenza, 2019; Colgan et al., 2020; Lee et al., 2020; Chen and Gaber, 2021), knowledge of hypoxia-regulated pathways in lymphedema will deepen our understanding of this disease. Here we review how hypoxic stress may influence key lymphedema pathology.

### Hypoxia and Hypoxia-Inducible Factors

Oxygen homeostasis is an organizing principle for understanding human physiology and pathobiology (Semenza, 2012). Hypoxia reflects conditions in which oxygen demand exceeds supply (Cavadas et al., 2013). The most common causes of tissue hypoxia include vascular insufficiency, edema, and inflammation (Lee et al., 2020). Mammals have evolved systemic adaptive mechanisms to cope with hypoxic stress by increasing ventilation, cardiac output, new vessel growth, and circulating red blood cell numbers (Lee et al., 2020). In contrast, edema and inflammation-associated hypoxia discretely influence the bioenergetics of various cell types within affected tissues and regulate disease pathogenesis (Poeze, 2006; Eltzschig and Carmeliet, 2011; Palazon et al., 2014; Colgan et al., 2020). The best-studied mechanism of responses to hypoxia involves hypoxia-inducible factors (HIFs). HIFs are heterodimers comprised of two subunits: the constitutively expressed β subunit (HIF-1β) and one of the α subunits (HIF-1α or HIF-2α) (Prabhakar and Semenza, 2015; Lee et al., 2020). Under normoxic conditions, the α subunits are subject to proteasome-mediated degradation involving enzymatic activities of dioxygenases termed prolyl hydroxylases (PHDs) and the von Hippel-Lindau tumor suppressor protein (pVHL) in the presence of ferrous iron (Fe^2+^) and α-ketoglutarate (Semenza, 2012; Lee et al., 2020). Reduced oxygen availability leads to HIF-α stabilization through mechanisms involving paradoxically increased production of reactive oxygen species (ROS) by complex III of the electron transport chain in mitochondria, as well as increased production of L-2-hydroxyglutarate (L-2HG), succinate, and fumarate (Nathan and Cunningham-Bussel, 2013; Lee et al., 2020). Activated HIF-αs govern a variety of biological processes through transcriptional regulation of several thousand downstream targets that are responsible for cell differentiation, survival, and metabolic adaptation (Keith et al., 2011; Lee et al., 2020). HIF-1α and HIF-2α share about 48% amino acid identity and regulate both overlapping and distinct downstream genes (Tian et al., 1997; Majmundar et al., 2010). Notably, HIF-1α is more active in conditions with intense hypoxia, while HIF-2α functions more prominently under mild or physiological hypoxia (Koh and Powis, 2012) or at homeostasis (Jiang et al., 2019b; Pasupneti et al., 2020).

### HIF and Lymphangiogenesis in Lymphedema

The evolution of secondary lymphedema involves early regenerative lymphangiogenesis following lymphatic injury and late lymphatic remodeling when the disease progresses (Rutkowski et al., 2006; Mihara et al., 2012). Evidence from both preclinical and clinical studies indicates that lymphedema tissues are hypoxic with metabolic derangement (Tabibiazar et al., 2006; Zampell et al., 2012a; Jiang et al., 2020). Lymph stasis and inflammation stabilize HIF-1α in mouse lymphedema, and this high tissue HIF-1α expression is required for reparative lymphangiogenesis and wound healing (Zampell et al., 2012a). Mechanistically, HIF-1α induces LEC (lymphatic endothelial cell) VEGFC and VEGFR3 expression and promotes lymphangiogenesis (Min et al., 2011; Zampell et al., 2012a; Han et al., 2019). LEC HIF-1α deletion transiently aggravates edema in a mouse-tail lymphedema model, corroborating a critical role of LEC HIF-1α in lymphatic regeneration immediately post-surgery (Jiang et al., 2020). By contrast, lymphatic HIF-2α plays a crucial role in embryonic lymphatic development, adult lymphatic maintenance, and lymphatic regeneration in lymphedema by activating TIE2 signaling, which is required for lymphatic vascular maturation and maintenance (Dellinger et al., 2008; Kajiya et al., 2012; Kim et al., 2017; Jiang et al., 2020). Interestingly, lymphatic HIF-2α decreases in lymphedematous tissues, a phenomenon likely attributable to heightened IFN-γ and HIF-1α expression (Takeda et al., 2010; Meneses and Wielockx, 2016; Jiang et al., 2020; Becker et al., 2021). In blood vasculature, HIF-2α is an endothelial survival factor (Jiang et al., 2019b; Pasupneti et al., 2020). HIF-2α also regulates vascular maturation and stabilization (Skuli et al., 2012), whereas HIF-1α tends to promote vascular sprouting (Koh and Powis, 2012). Further, HIF-2α regulates the expression of lymphatic lineage marker Prox-1 (Zhou et al., 2013). Therefore, increased HIF-1α expression with concomitant HIF-2α reduction in LECs may exacerbate lymphatic malfunctioning by promoting non-productive lymphangiogenesis and LEC phenotypic transition in chronic stage lymphedema.

### HIF and Inflammation in Lymphedema

Hypoxia and inflammation often coexist in pathological conditions (Bartels et al., 2013; Taylor and Colgan, 2017; Colgan et al., 2020). For example, increased immune cell infiltration in inflammatory loci increases oxygen consumption, disrupting the balance between demand and supply of O_2_, consequently promoting hypoxia (Eltzschig and Carmeliet, 2011). Conversely, hypoxia and HIFs promote immune dysregulation, which drives tissue dysfunction and disease progression; the degree of hypoxia determines the extent to which HIF activation in immune cells and subsequent downstream effects (Taylor and Colgan, 2017). Hypoxia modulates both innate and adaptive immunity (Taylor and Colgan, 2017). The coevolving of hypoxia and inflammation is a feature of various diseases such as inflammatory bowel disease, rheumatoid arthritis, and acute lung injury (Watts and Walmsley, 2019). Hypoxia and inflammation also appear to influence lymphedema pathogenesis (Jiang et al., 2020).

HIFs control macrophage differentiation. Specifically, HIF-1α promotes macrophage differentiation into an M1 lineage, while HIF-2α fosters M2 polarization (Imtiyaz et al., 2010; Takeda et al., 2010; Domblides et al., 2018). Macrophages may differentiate primarily into the M2 phenotype in lymphedema (Ghanta et al., 2015). However, macrophages localized in fat tissue within lymphedematous skin display the M1 phenotype (Tashiro et al., 2017). Divergent macrophage differentiation in different tissue compartments may explain why macrophages play protective or harmful roles in lymphedema in a context-dependent manner (Kataru et al., 2009; Ginhoux and Jung, 2014; Ghanta et al., 2015). We showed that myeloid cell-specific HIF-2α deficiency aggravates tissue swelling following lymphedema surgery (Jiang et al., 2020), suggesting M2 macrophages may promote lymphatic repair and alleviate edema in subacute mouse-tail lymphedema. More in-depth characterization of HIF isoform expression in macrophages during the acute and chronic phases of lymphedema will enhance our understanding of how HIFs modulate macrophage differentiation and regulate lymphedema pathology. Future investigation of macrophage biology in lymphedema should also go beyond the overly simplified M1, M2 dichotomy (Ginhoux and Jung, 2014).

HIF-1α controls the balance of Th17/Treg cell differentiation (Dang et al., 2011; Hsiao et al., 2015; Tao et al., 2015; Clever et al., 2016). Specifically, HIF-1α induces the expression of the transcriptional factor RORγT and the Th17 signature gene IL-17A that requires STAT3 activation; concomitantly, HIF-1α promotes proteasome-mediated degradation of FOXP3, the key factor for Treg differentiation (Dang et al., 2011; Darce et al., 2012). HIF-1α also favors Th17 over Treg differentiation by skewing T cell glycolytic metabolism (Shi et al., 2011). HIF-2α, by contrast, is crucial for Treg stability and function, and this regulation may partly depend on suppressing HIF-1α gene transcription and protein accumulation (Hsu et al., 2020). It is worth noting that HIF-1α may also promote Treg differentiation and play a role in suppressing mucosal inflammation (Clambey et al., 2012). While hypoxia results in a shift toward Th2 response (Lederer et al., 1999), it is not entirely clear how HIF isoforms may differentially regulate Th1 and Th2 differentiation. CD4^+^ T cell expansion is a prominent pathology in lymphedema (Ly et al., 2017; Jiang et al., 2018; Azhar et al., 2020). HIF isoforms, therefore, could conceivably modulate the differentiation and function of different immune compartments in lymphedema.

### HIF and Adipose Expansion in Lymphedema

Hypoxia plays a pathogenic role in obesity (Norouzirad et al., 2017). Hypoxia induces adipocyte death and consequent macrophage recruitment (Cinti et al., 2005; Yin et al., 2009); these infiltrating cells, regulated primarily by HIF-1α, play a crucial role in initiating obesity-related disorders, including metabolic reprogramming, insulin resistance, and inflammation (Mazzatti et al., 2012). Accordingly, adipocyte-specific HIF-1α deletion improves insulin sensitivity and diminishes adipose mass in high-fat diet-fed mice (Jiang et al., 2011). These studies demonstrated that adipocyte expression of HIF-1α promotes obesity and related pathologies. By contrast, adipocyte HIF-2α protects against metabolic maladaptation, insulin resistance, and inflammation in diet-induced obesity (García-Martín et al., 2016). Aberrant fat accumulation and metabolism in lymphedema resemble that of obesity (Mehrara and Greene, 2014). For instance, lymphedema patients exhibit increased serum levels of adipokines such as adiponectin and leptin (Zaleska and Olszewski, 2017). Lymphedema tissues also express increased proinflammatory cytokines such as TNF-α, IL-6, and IL-1β (de Ferranti and Mozaffarian, 2008; Jiang et al., 2018). In particular, high IL-6 expression appears to associate with fat deposition in both lymphedema and obesity (Olszewski et al., 1992; Mohamed-Ali et al., 1997; Fried et al., 1998; Cuzzone et al., 2014). Hypoxia also influences adipose pathology through modulating the differentiation and function of infiltrating macrophages (Engin, 2017). Adipose tissue hypoxia promotes proinflammatory M1 macropahge differentiation and strengthens the production of IL-6, IL-1β, and iNOS (Fujisaka et al., 2013). In addition, hypoxic macropahges in an environment with increased saturated free fatty acids also enhance adipocyte IL-6 and MCP-1 production (Snodgrass et al., 2016). Whether HIF-α isoforms similarly regulate fat accumulation in lymphedema as observed in the context of obesity is an open question. Interestingly, in visceral adipose tissue (VAT), estrogen signaling modulates stromal cell expression of the HIF-1α target gene CD73 (Synnestvedt et al., 2002), which leads to a divergent Treg population in female adipose tissue compared to males (Vasanthakumar et al., 2020). These studies support the notion that hypoxia may regulate adipose tissue inflammation in a sexually dimorphic manner. This concept can be used to explain why lymphedema preferentially develops in females (Trincot and Caron, 2019).

### HIF and Fibrosis in Lymphedema

Fibrosis, characterized by excessive deposition of extracellular matrix (ECM) components such as collagen and fibronectin, is a dysregulated tissue repair response following various types of tissue injury with chronic inflammation (Henderson et al., 2020). Fibroblasts are the critical source of excessively produced ECM in remodeled fibrotic tissue (Henderson et al., 2020). A recent single-cell RNA-Seq analysis of keloid and scleroderma samples identified four subpopulations of skin fibroblasts: secretory-papillary, secretory-reticular, mesenchymal, and proinflammatory; among which the percentage of mesenchymal fibroblast is the primary fibroblast subtype involved in skin fibrosis (Deng et al., 2021). Fibrotic tissues often display features of chronic hypoxia, and hypoxia-regulated signaling regulates collagen production, accumulation, and crosslinking (Gilkes et al., 2013; Gilkes et al., 2014; Xiong and Liu, 2017). Hypoxia promotes fibroblast transition to a myofibroblast-like phenotype and skin fibrosis through HIF-1α-mediated activation of the TGF-β and NF-κB signaling pathways (Distler et al., 2007; Zhao et al., 2017; Lei et al., 2019). Intriguingly, HIF-1α-mediated fibroblast to myofibroblast differentiation depends on augmented glycolysis and increased succinate production (Xie et al., 2015). Specifically, elevated lactate levels resulting from augmented fibroblast glycolytic metabolism stimulate TGF-β signaling by reducing extracellular pH and activating latent TGF-β (Kottmann et al., 2012). HIF-1α may also enhance myofibroblast collagen production by inducing glutaminase 1 (GLS1) expression and promoting glutaminolysis (Ge et al., 2018; Xiang et al., 2019; Bouthelier and Aragonés, 2020). Collectively, HIF-1α-dependent hypoxic responses foster myofibroblast differentiation and ECM protein production by reprogramming metabolism and activating the TGF-β pathway (Gibb et al., 2020). Studies have also demonstrated that Th2 and Th17 cells and macrophages are involved in tissue fibrosis (Barron and Wynn, 2011; Wynn and Vannella, 2016; Vannella and Wynn, 2017; Henderson et al., 2020). Therefore, HIF isoforms may regulate fibrotic remodeling by controlling the differentiation of CD4^+^ T helper cells and macrophages.

Fibrosis in lymphedema affects the dermis and subcutaneous tissue, representing a crucial factor promoting tissue hardening and non-pitting edema (Rutkowski et al., 2010; Zampell et al., 2012c; Gardenier et al., 2016; Tashiro et al., 2017). Additionally, diffuse tissue fibrosis worsens lymphatic dysfunction by directly impairing lymphatic vascular regeneration (Lynch et al., 2015). Collecting lymphatic vessels also undergo fibrotic remodeling, including excessive collagen deposition and disorganized expansion of the smooth muscle cell layer; these structural changes decrease lymphatic vessel contractility, aggravate drainage deficiency, and increase lymph leakage (Olszewski, 2002; Mihara et al., 2012; Gardenier et al., 2016). Currently, studies investigating hypoxia in promoting fibrosis in lymphedema are limited. Knowledge on how hypoxia may influence the phenotype of lymphatic vascular cells and enhance fibrotic tissue remodeling will improve our understanding of molecular pathways involved in lymphedema progression.

## Concluding Remarks

**FIGURE 1:**
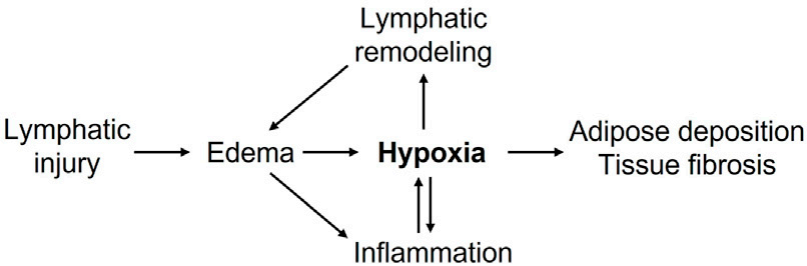
Putative roles of hypoxia in promoting lymphedema pathogenesis. Lymphatic injury-caused edema promotes inflammation and tissue hypoxia. Decreased tissue oxygenation further enhances inflammatory responses, lymphatic vascular remodeling, adipose deposition, tissue fibrosis, and collectively exacerbates lymphedema.

Chronic lymphedema poses a significant health burden on afflicted patients and requires effective pharmacological treatments. Hypoxia and HIF-mediated responses appear to regulate lymphedema pathogenesis (Figure 1). Available data concerning the roles of HIF isoforms in lymphangiogenesis, inflammation, fatty expansion, and fibrotic tissue remodeling suggested a possible opposing role of HIF isoforms, with HIF-2α possibly playing beneficial effects in lymphedema. A fuller exploration of hypoxia-regulated molecular pathways in lymphatic endothelial, immune, and stromal compartments will enhance our understanding of lymphedema pathogenesis and likely provide novel therapeutic targets to improve lymphatic repair, adipose expansion, and fibrotic remodeling in lymphedema.
